# Increasing Smoking Cessation Adherence: Do We Need to Consider the Role of Executive Function and Rumination?

**DOI:** 10.5964/ejop.v16i1.2279

**Published:** 2020-03-03

**Authors:** Marianna Masiero, Mark Cropley, Gabriella Pravettoni

**Affiliations:** a Applied Research Division for Cognitive and Psychological Science, European Institute of Oncology IRCCS, Milan, Italy; b Department of Biomedical and Clinical Sciences, University of Milan, Milan, Italy; c School of Psychology, Faculty of Health and Medical Sciences, University of Surrey, Surrey, United Kingdom; d Department of Oncology and Hemato-Oncology, University of Milan, Milan, Italy

**Keywords:** rumination, smoking, executive functions, decision-making, personality

## Abstract

Despite the cost and health consequences, a large number of people continue to smoke cigarettes worldwide every day. Notwithstanding, there have been a number of interventions to help people stop smoking but, in general, these have produced only limited success, and better interventions are needed. Accruing evidence affirmed that rumination and executive function play a pivotal role in cigarette smoking behavior, and in this editorial, we describe and discuss the key findings between these constructs and smoking, and argue that an impairment in executive functions does not act alone, but interacts with rumination by directing attention to depressive thoughts, thereby reducing the ability of smokers to engage in constructive behaviors, such as quitting smoking. Finally, we offer a new theory-driven model based on a deep understanding of the interactions between executive functions and rumination and potential moderator effects.

Tobacco cigarette smoking is a grave epidemic that will cause more than 6-8 million deaths before 2030 (WHO, 2018). Notwithstanding, a large number of people continue to smoke and worldwide, every day, and approximately 2000 young people will smoke their first cigarette, with many of these becoming habitual smokers (Lewis et al., 2019). There have been many treatments to help people quit smoking, yet these have not, on the whole proved effective. Generally, smoking cessation interventions (referring to intervention based on pharmacotherapy, both Bupropion and/or Varenicline, combined with cognitive-behavioral support) produce moderate success rates and these fall substantially at one year, as the majority of individuals will relapse (Hayes, Jackson, Dickinson, & Miller, 2018; Lucchiari, Masiero, Botturi, & Pravettoni, 2016; Masiero, Riva, Fioretti, & Pravettoni, 2016; Oliveri, Renzi, Masiero, & Pravettoni, 2015). The low effectiveness of these treatments is probably due to the low motivation to maintain abstinence after the interruption (Perski, Herd, Brown, & West, 2018; Piñeiro et al., 2016). It is therefore of importance to develop new theory-driven models in order to explain the psychological mechanisms acting as roadblocks to motivation to quit or relapse. Two psychological constructs that may potentially be informative are executive function (EF) and rumination.

Accruing evidence has supported the role of rumination (Riley, Park, & Laurenceau, 2019) and EF (Allan, McMinn, & Daly, 2016) as potential factors able to explain the complexity of the adoption of health risk behaviors such as cigarette smoking. Nevertheless, it is not clear how rumination and EF affect the adoption of the smoking behavior and/or the motivation to quit, as research conducted until now tends to be limited by methodological and theoretical shortcomings. Particularly, Fox and colleagues (2017) stressed the tendency to use different types of methodologies to assess EF, and for each of them to assess a specific component of EF for example, Wisconsin Card Sorting Test (set-shifting), Stroop Test (response inhibition), Go/No-go task (attention and set-shifting), Trail Making Test (visual attention), etc. with research often providing mixed results. For example, Ernst and colleagues did not find differences between smokers, former smokers and never smokers in EF (Ernst, Heishman, Spurgeon, & London, 2001), but others have founded significant differences (Campos, Serebrisky, & Castaldelli-Maia, 2016; Glass et al., 2009) . Furthermore, rumination and EF are typically studied separately (Glass et al., 2009; Stautz, Pechey, Couturier, Deary, & Marteau, 2016) and/or in association with other psychological factors, for example, anxiety, depression, emotion perception (referring to the capacity to correctly recognize facial expressions associated to the emotions etc.) (Fox et al., 2017; Meyers et al., 2015; O’Loughlin et al., 2017). Moreover, research that has examined the role of rumination in smokers has mainly focused on people having depressive disorders (Herrera & McChargue, 2011). Finally, there are only a limited number of studies that have investigated a possible interaction between rumination and cognitive function, reporting that ruminative thoughts interfere with the EF (Watkins & Brown, 2002) by reducing the ability to inhibit stimuli (Lyubomirsky, Tucker, Caldwell, & Berg, 1999) or task switching (Gustavson et al., 2019; Whitmer & Banich, 2007). All of these studies are based on general ruminative negative thoughts relating to cravings, urgency for cigarettes and perception of negative emotional arousal; none of them reported a specific focus on aptitude to ruminate on previous failed attempts to quit.

## Ruminative and Non-Ruminative Cigarette Smokers

Perseverative cognition, worry, and rumination, are strongly associated with the adoption of health-risk behaviours but in different directions (Clancy, Prestwich, Caperon, & O’Connor, 2016). Worry being characterized by negative and uncontrollable thoughts and images; while rumination is defined as experiencing repetitive, intrusive and negative thoughts (Querstret & Cropley, 2013). Whereas worry supports the adoption of healthy behaviors, rumination impedes them. McCaul and colleagues (2007) pointed out that smokers exposed to negative stimuli of cigarette-related material (e.g. pictures and statements), reported a higher worry, related to effects of smoking on their health, driving them to made factual plans to quit (McCaul, Mullens, Romanek, Erickson, & Gatheridge, 2007). In opposition, increased ruminative thought is associated with the adoption or engagement of unhealthy behaviors, such as unhealthy diet, smoking, alcohol consumption and a broad substance abuse (Allan et al., 2016; Cropley et al., 2012; Nosen & Woody, 2014; Sperlich & Maina, 2014). Nosen and Woody observed that motivated smokers who ruminate in response to negative affect, craving, and withdrawal, are at a greater risk to be continuous smokers and to make a briefer duration of cessation attempts (Nosen & Woody, 2014). In addition, a cross-sectional study with 3129 single smoking mothers reported that the tendency to ruminate increased the number of daily cigarettes smoked (Sperlich & Maina, 2014). It has been argued that rumination fosters attention on depressive thoughts, distracting smokers to (Querstret & Cropley, 2012) engage in other constructive behaviors such as quitting smoking (Lyubomirsky, Kasri, Chang, & Chung, 2006). For example, in lung cancer patients who smoke, rumination concerning past decisions (“*Why have I smoked?*” - “ *It was the worst decision of my life*” - “*If I could come back and change my actions*” etc.) increases depressive symptomatology (Criswell, Owen, Thornton, & Stanton, 2016), and consequently reduces motivation and impedes the quitting attempts (Lucchiari et al., 2016; Masiero, Renzi, Mazzocco, & Pravettoni, 2019; Masiero, Lucchiari, et al., 2019). Further, rumination has adverse effects on thinking and concentration, causing motivational deficits, which may inhibit the ability to take appropriate actions to resolve problems (Lyubomirsky et al., 1999).

## Individual Psychological Patterns in Ruminative Cigarette Smokers

The impairment in inhibition responses is partially moderated by personality traits such as impulsiveness. Indeed, impulsive and ruminating smokers find it more difficult to stop smoking and their smoking trajectory is characterized by several failed quit attempts (Dvorak, Simons, & Wray, 2011). In particular, impulsiveness works through two channels: *emotional-oriented* (i) and *attentive-oriented* (ii): (i) negative emotions increase more quickly during a quit attempt in smokers, and in particular those with a higher level of non-planning impulsivity (VanderVeen, Cohen, Trotter, & Collins, 2008), thereby increasing the risk of relapse at three and six months (López-Torrecillas, Perales, Nieto-Ruiz, & Verdejo-García, 2014); (ii) high level of impulsiveness renders smokers more susceptible to smoking-related stimuli and to depressive rumination, increasing the risk to consume more daily cigarettes and relapse after a quit attempt (Clancy et al., 2016; Herrera & McChargue, 2011). In this way, rumination and smoking reinforce each other, creating a “vicious circle” thereby reducing the motivation to quit (Richmond, Spring, Sommerfeld, & McChargue, 2001). It is likely that ruminating about unpleasant thoughts connected with failed attempts to quit, increases emotional distress in smokers, and reinforces a loss of control and a reduction in self-efficacy needed to successfully quit (Etter, Mohr, Garin, & Etter, 2004). Strong self-control and a high self-efficacy are key predictors for resisting cravings and for avoiding potential smoking-related stimuli during withdrawal (Daly, Egan, Quigley, Delaney, & Baumeister, 2016). Evidence reported above is consistent with the Self-Regulatory Executive Function (S-REF) theory (Wells, 2000, 2008) and Perseverative Cognition Hypothesis (PCH) (Brosschot, Gerin, & Thayer, 2006; Spada, Nikčević, Moneta, & Wells, 2007) in that cigarette smoking is used as a maladaptive coping strategy to reduce rumination and to regulate their negative emotions (Riley et al., 2019; Sperlich & Maina, 2014).

## Impairment in Executive Functions Along Smoking Trajectory

EFs is an “*umbrella term*” used to refer to a set of psychological processes supporting goal-directed behaviors but there is a lack of an accepted definition within the literature. Wilens and colleagues (2011) suggested to split EFs in three main mechanisms: (i) intention to inhibit a response or to defer it over time; (ii) action planning; and (iii) mental representation of each task (Wilens et al., 2011). Preserved EFs permit individuals to plan and filter competing information for achieving a specific goal, avoiding distraction and inhibiting goal-inconsistent responses (Hollen et al., 2013). Otherwise, an impairment in EFs seems to be associated with unhealthy behaviours such as drinking alcohol, cigarette smoking, and eating high-fat foods (Stautz et al., 2016). Particularly, EFs are key factors in the modulation of smoking behaviour (Almeida et al., 2011; Campos et al., 2016; Jacobsen et al., 2005; Lyvers, Maltzman, & Miyata, 1994; Mons, Schöttker, Müller, Kliegel, & Brenner, 2013; Ott et al., 2004; Sabia et al., 2012). Campos and colleagues observed that abstinence smokers had a low performance on psychomotor, memory and attentional tasks during nicotine withdrawal, and the impairment persisted after quitting for a few months (Campos et al., 2016). Normally, an impairment in EFs in smokers arises only in adulthood, between 43 and 53 years, after a prolonged exposure to nicotine properties’. It is suggested that there is a “*temporal window*”, in which the deficit progressively arises (Campos et al., 2016). Smokers with preserved EF are more likely to remain abstinent 24 months following a quit attempt, compared to smokers with an impairment in EF (Almeida et al., 2011) and the worsening of EF were more important in habitual smokers (Mons et al., 2013; Ott et al., 2004; Sabia et al., 2012). Furthermore, Glass and colleagues (Glass et al., 2009) reported that chronic consumption of cigarettes is correlated with lower neurocognitive performance in different cognitive functions, such as working memory (Jacobsen et al., 2005), psychomotor speed, verbal memory and visual search (Glass et al., 2009) cognitive flexibility (Kalmijn, Van Boxtel, Verschuren, Jolles, & Launer, 2002). These data were supported experimentally by means of the Wisconsin Card Sorting Test (WCTS), where heavy smokers reported more errors and perseverative responses in WCTS compared to never smokers and former smokers (Lyvers et al., 1994).

Moreover, the abstinence period might modulate the impairment in EFs. For example, recent former smokers showed greater cognitive impairment in EFs compared with long-term former smoker (Lyvers et al., 1994; Sabia et al., 2012). However, long-term former smoker’s cognitive ability was similar to individuals who never smoked (Sabia et al., 2012). This suggests that the age and exposure period to nicotine’s properties have an influence on impairment in EFs. In particular, it has been observed that during adolescence an early exposure to nicotine leads to higher levels of dependency by exerting neurotoxic effects in the prefrontal cortex (PFC) interfering with cognitive development, executive functioning, and inhibitory control (Goriounova & Mansvelder, 2012). In general, smokers who begin to smoke at an older age are less impaired than smokers who begin to smoke at younger ages (Allan et al., 2016; Jacobsen et al., 2005; Mons et al., 2013). Besides, the age of smoking onset is associated with a weakened working memory and reduced information processing ability (Ernst et al., 2001). Furthermore, adult smokers who started smoking at a young age (roughly 13 years old) performed worse compared with adult smokers who started smoking at a later age. This higher vulnerability to impairment in EFs in smokers that start earlier may be due to early stages of brain maturation, having a more strong effect on executive functions such as inhibitory control and sustained attention (Mashhoon, Betts, Farmer, & Lukas, 2018). Considering the detrimental effects smoking has on cognition, particularly in those who are exposed to nicotine at an early age, and the moderate success rates of current smoking cessation programmes, it is imperative to develop and examine innovative ways to tackle this problem.

## Assessing the Combined Effect of Rumination and EF Impairment on Motivation to Interrupt

EFs have a key role in planning, sequencing and monitoring goal-directed thoughts, behaviours and emotions (Allan et al., 2016; Hofmann, Schmeichel, & Baddeley, 2012; Razani, Boone, Lesser, & Weiss, 2004), and alteration of higher cognitive processes, for example, self-regulation and planning (Fox et al., 2017), reduce the probability to achieve personal goals. In line with Meyers and colleagues (2015), emotion processing and executive function decrements may interfere with an individual's ability to quit smoking (Meyers et al., 2015). Currently, a number of studies (Glass et al., 2009; Mons et al., 2013; Sabia et al., 2012; Wilens et al., 2011) supports the view that smoking has detrimental effects on EFs in habitual smokers, modulating the motivation to interrupt. Similarly, ruminating smokers have a greater risk to be continuous smokers and/or to make several failed quit attempts (Dvorak et al., 2011; Lyubomirsky et al., 2006; Nosen & Woody, 2014).

**Figure 1:**
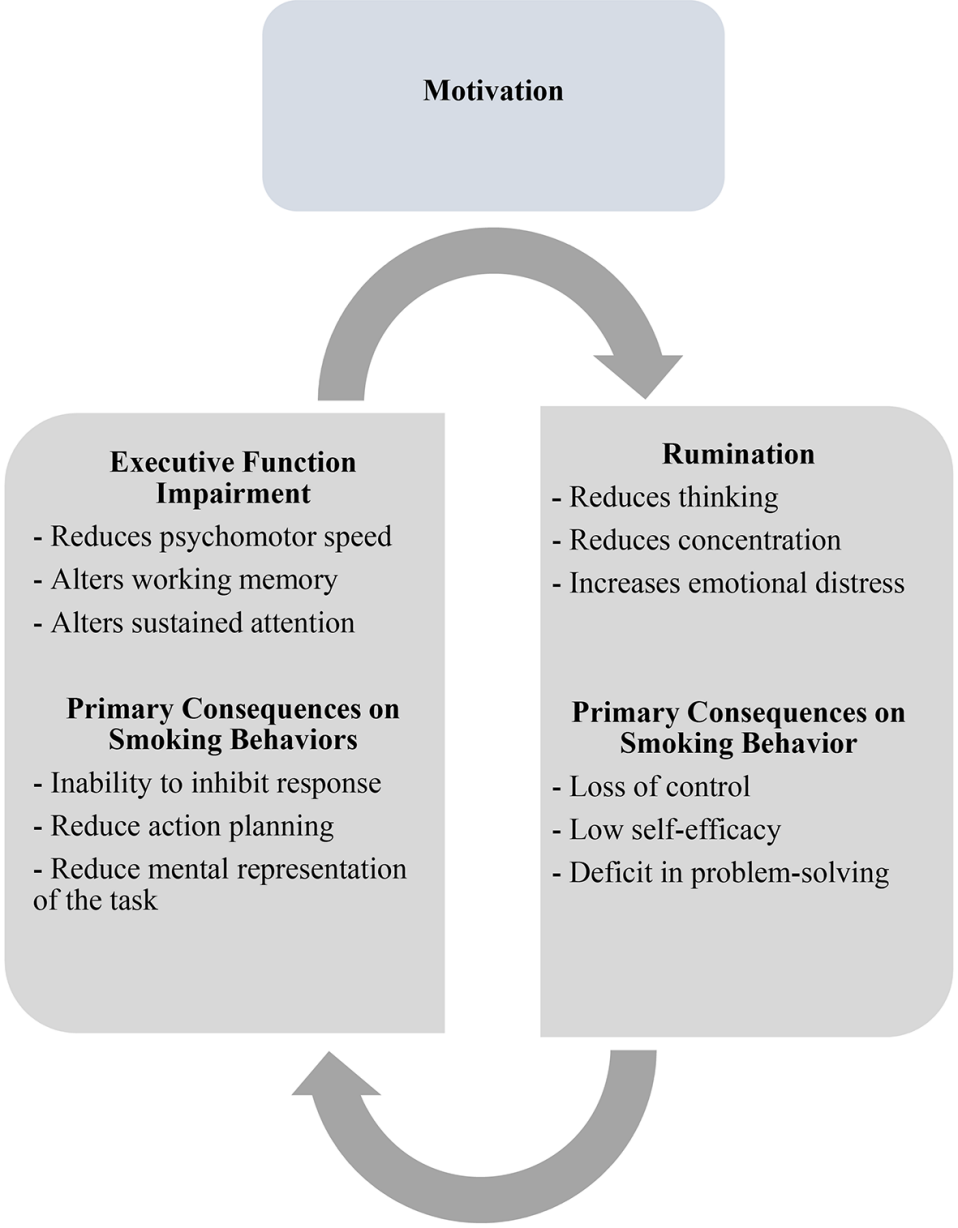
Theory driven model able to explain interaction between EF and rumination. *Note.* An impairment in EF causes an alteration in psychomotor speed, working memory and sustained attention that provokes an imbalance in capacity of inhibit irrelevant response, reduces action planning and mental representation of the task. This affects negatively motivation to quit, reducing in smokers the capacity to plan goal direct behaviors. This impairment may interact with the rumination that reduces thinking, concentration and increases emotion distress causing in the individual a loss of control, a reduction in his/her self-efficacy, and an important deficit in problem-solving strategies related to the interruption.

Notwithstanding, we argue that a real comprehensive theory-driven model able to explain psychological mechanisms underlying motivation to quit, should be based on a deep understanding of interaction between EFs and rumination, and potential moderator effects (Figure 1).

In particular, we reason that an impairment in EFs does not act alone, but interacts with rumination by directing attention to depressive thoughts, thereby reducing the ability of smokers to engage in other constructive behaviors such as quitting smoking (Lyubomirsky et al., 2006). In addition, the quit process may be affected by the propensity of smokers to ruminate on depressive thoughts, and past history of failed attempts to quit. Such ruminative thoughts increase the perception of loss of control, thereby reducing self-efficacy, which is considered pivotal in the motivation to quit (Schwarzer, 2008; Strecher, McEvoy DeVellis, Becker, & Rosenstock, 1986). Indeed, in line with Prochaska and DiClemente’s model (Prochaska & DiClemente, 1983), high self-efficacy and motivation to quit favour the transition from pre-contemplation (when smoker consider the possibility to quit) to preparation (when smoker did a real attempt to quit).

In addition, prolonged exposure to nicotine causes significant impairments in EFs, in particular, on the capacity to maintain an adequate mental set able to achieve future goals and inhibiting goal-inconsistent responses. This impairment in EFs may cause a significant reduction in the motivation to quit and reduce the likelihood of sustained abstinence.

Future studies should investigate how deficits in EFs and rumination interact, and how they reduce the motivation to quit cigarette smoking. Overall, we have identified three issues, that could/should be addressed by future research.

Firstly, how an impairment in EF in chronic cigarette smokers may reduce their action planning and mental representation of the tasks needed to quit smoking; and in which way are these processes associated with perseverative rumination about unpleasant thoughts related to an individual’s inability to quit.

Secondly, individual psycho-cognitive and behavioral patterns should be identified; as previously reported, the interaction between EF, rumination and motivation might be modulated by personality traits, such as impulsivity and physiological aspects related to the nicotine dependence, as well, the level of dependence, the past history of smoking and the number of daily cigarette.

Thirdly, the new line of research should be directed to enhance knowledge and our understanding of the psychological mechanisms affecting motivation and intention to quit, particularly in high risk populations such as patients with smoking related disease and/or heavy smokers enrolled in screening programs for early detection on lung cancer (Masiero, Renzi, Mazzocco, & Pravettoni, 2019). In particular in cancer population, where the neurotoxic effects of chemotherapy may cause a deficit in EF (Dietrich, Prust, & Kaiser, 2015), that might be associated with chronic cigarette smoking, and drastically reducing the attempts to quit.

A more detailed understanding could be the key to developing advanced smoking cessation programmes. We think that more research is needed to increase our understanding of these areas (i.e. rumination and executive function), and with “greater knowledge” we would be better placed to train health professionals who work within smoking cessation.
